# Association of Sarcopenic Dysphagia with Underlying Sarcopenia Following Hip Fracture Surgery in Older Women

**DOI:** 10.3390/nu12051365

**Published:** 2020-05-10

**Authors:** Ayano Nagano, Keisuke Maeda, Akio Shimizu, Shinsuke Nagami, Naohide Takigawa, Junko Ueshima, Masaki Suenaga

**Affiliations:** 1 Department of Nursing, Nishinomiya Kyoritsu Neurosurgical Hospital, 11-1 Imazuyamanaka-cho, Nishinomiya, Hyogo 663-8211, Japan; aya.k.nagano@gmail.com; 2 Department of Palliative and Supportive Medicine, Aichi Medical University Hospital, 1-1 Yazakokarimata, Nagakute, Aichi 480-1195, Japan; 3 Department of Nutrition, Hamamatsu City Rehabilitation Hospital, 1-6-1, Wago-kita, Naka-ku, Hamamatsu, Shizuoka 433-8127, Japan; a.shimizu.diet@gmail.com; 4 Department of Sensory Science, Faculty of Health Science and Technology, Kawasaki University of Medical Welfare, 288 Matsushima, Kurashiki, Okayama 701-0193, Japan; shinsuke.nagami.0514@gmail.com; 5 Department of Orthopedic Surgery, Nishinomiya Kyoritsu Neurosurgical Hospital, 11-1 Imazuyamanaka-cho, Nishinomiya, Hyogo 663-8211, Japan; takky@pq7.so-net.ne.jp; 6 Department of Clinical Nutrition and Food Service, NTT Medical Center, 5-9-22 Higashi-Gotanda, Shinagawa-ku, Tokyo 141-8625, Japan; j.ueshima@gmail.com; 7 Okinawa Chuzan Clinical Research Center, Chuzan Hospital, 6-2-1 Matsumoto, Okinawa 904-2151, Japan; suemasa0916@gmail.com

**Keywords:** acute care, hospitalization, sarcopenia, swallowing disorders, dysphagia, malnutrition

## Abstract

This study aimed to investigate the association between the development of dysphagia in patients with underlying sarcopenia and the prevalence of sarcopenic dysphagia in older patients following surgical treatment for hip fracture. Older female patients with hip fractures (*n* = 89) were studied. The data of skeletal muscle mass, hand-grip strength, and nutritional status were examined. The development of dysphagia postoperatively was graded using the Food Oral Intake Scale by a certified nurse in dysphagia nursing. The patients’ mean age was 85.9 ± 6.5 years. The prevalence of sarcopenia was 76.4% at baseline. Of the 89 patients, 11 (12.3%) and 12 (13.5%) had dysphagia by day 7 of hospitalization and at discharge, respectively. All patients who developed dysphagia had underlying sarcopenia. Lower skeletal muscle mass index (SMI) (<4.7 kg/m^2^) and grip strength (<8 kg) at baseline indicated a higher incidence of dysphagia on day 7 (*p* = 0.003 and Phi = 0.391) and at discharge (*p* = 0.001 and Phi = 0.448). Dysphagia developed after hip fracture surgery could be sarcopenic dysphagia, and worsening sarcopenia was a risk factor for dysphagia following hip fracture surgery. Clinicians and medical coworkers should become more aware of the risks of sarcopenic dysphagia. Early detection and preventive interventions for dysphagia should be emphasized.

## 1. Introduction

Recently, the association between sarcopenia and dysphagia has received increased research attention. Maeda et al. reported that 76.8% and 30% of hospitalized older patients had sarcopenia and dysphagia, respectively [1]. This study further revealed that in the presence of sarcopenia, the ability to perform activities of daily living (ADL, assessed by Barthel Index) and the skeletal muscle mass index (SMI) were independent risk factors for dysphagia [1]. Sarcopenic dysphagia is a condition characterized by sarcopenia-induced swallowing disorder and the loss of swallowing muscle mass and function. Four Japanese professional organizations consolidated the currently available evidence in this area in the position paper report in 2019 [2]. In addition, Mori et al. proposed a diagnostic flowchart for sarcopenic dysphagia, and its reliability and validity have been verified [3]. Sarcopenic dysphagia is diagnosed when swallowing disorder is present with whole-body sarcopenia except for the presence of obvious causative disease of dysphagia such as neurological diseases. Wakabayashi et al. reported that sarcopenia was the possible cause of sarcopenic dysphagia in 32% of inpatients who underwent dysphagia rehabilitation and that patients with dysphagia due to sarcopenia had a worse prognosis than patients with dysphagia due to other diseases [4]. Previous studies have shown that dysphagia may develop in older patients after the onset of acute diseases such as pneumonia [5], or after undergoing rehabilitation [6], or in cases of sarcopenia-related conditions such as low physical function and malnutrition [5,7]. In addition, high-dose chemotherapy and radiotherapy for head and neck cancer can cause sarcopenic dysphagia [8,9,10]. However, the prevalence of sarcopenic dysphagia has received little attention up to now. 

Hip fractures commonly present with sarcopenia. Cases of sarcopenia were reported in 17~58% of older patients with hip fractures [11,12,13]. Furthermore, dysphagia was reported to occur in 5.3~44% of patients who underwent operative treatment for hip fractures [14,15,16,17]. Dysphagia in older patients with hip fracture was associated with risk factors such as low serum albumin levels, female gender, confusion after surgery, and preoperative worsening of physical status [14,15,16,17]. In particular, no study has yet explored the association between sarcopenia and dysphagia following hip fracture.

It was hypothesized that dysphagia development in older patients after hip fracture surgery is associated with sarcopenia or sarcopenia-related factors (muscle mass and hand-grip strength). Therefore, this study was conducted to investigate the association between underlying sarcopenia and postoperative dysphagia and the prevalence of sarcopenic dysphagia in older patients who sustained hip fractures and underwent surgical treatment.

## 2. Materials and Methods 

### 2.1. Ethics

This study was approved by the institutional review board of Chuzan Hospital (approval ID: 19-74). The consents were obtained by the opt-out method. The study complies with the principles stated in the Declaration of Helsinki [18].

### 2.2. Participants

The study design was single-center, observational, and retrospective. We collected data pertaining to consecutive female patients with hip fractures who were admitted at the orthopedic department in Nishinomiya Kyoritsu Neurosurgical Hospital in Japan between November 2018 and December 2019. Patients aged ≥65 years who had sustained fractures caused by falls and were treated surgically were included. Patients in whom the body composition and hand-grip strength could not be measured within 5 days following admission, those who had metallic device implantations before the injury, and patients who died or were discharged within 1 week after admission were excluded. Patients having dysphagia before the injury were also excluded from the analysis to study newly developed dysphagia after surgery. The patients with a Food Oral Intake Scale (FOIS) level of ≤5 were diagnosed with dysphagia at admission [1].

To investigate whether sarcopenia was associated with dysphagia development at day 7 and at discharge, SMI and hand-grip strength were measured at admission and were considered as baseline exposure factors. The Geriatric Nutritional Risk Index (GNRI) tool, which is an objective screening tool using serum albumin level, body weight, and height, was used to assess the nutritional status [19]. The calculation uses the following formula [19]:

GNRI = (1.489 × albumin (g/L)) + (41.7 × weight (kg)/ideal body weight)

Ideal body weight was calculated using the Lorentz formula as follows:

For women: height − 100 − [(height − 150)/2.5]

Nutrition-related risk categories were defined according to the GNRI values as major risk (GNRI: <82); moderate risk (GNRI: 82 to <92); low risk (GNRI: 92 to ≤98); no risk (GNRI: >98) [19]. Patients’ swallowing ability was monitored regularly by a nurse certified in dysphagia nursing (certified by the Japanese Nursing Association).

### 2.3. Baseline Parameters 

The weight was measured using a patient lift scale (Clear Lift Scale CLS-420, Bio International, Japan) and the height in the supine position. Skeletal muscle mass was measured by nurses of the orthopedic ward using the bioelectrical impedance analyzer (BIA) in the neutral spine position after the patient rested in this position for ≥15 min using a multifrequency BIA instrument (Inbody S10; Inbody, Tokyo, Japan). The SMI was calculated as a ratio between the appendicular skeletal muscle mass and the height squared. Likewise, physiotherapists measured the hand-grip strengths using the Smedley dynamometer (TTM-YD, Tsutsumi Industry, Japan) on the second day of rehabilitation. The women were in a sitting position during the measurements, and the higher of two measurements on the dominant hand was considered for the diagnosis. Based on the cutoff criteria stated by AWGS 2019 [20], women having SMI <5.7 kg/m^2^ and hand-grip strength <18 kg were diagnosed with sarcopenia. Gait speed or balance tests were not performed as our subjects could not walk due to fracture. 

Abilities to perform ADL at baseline and at discharge were evaluated with the Functional Independence Measure (FIM) [21], which is composed of 13 motor and 5 cognitive domains; each domain is scored from 1 (total assistance) to 7 (complete independence). A total FIM score ranges from 18 to 126, with higher FIM scores indicating higher independence in ADL. 

The subjects’ physical condition before surgery was assessed by ASA scores (American Society of Anesthesiologists) [14,22]. The ASA physical status classifies the patients’ fitness before surgery into 6 classes: (1) normal health, (2) mild systemic disease, (3) severe systemic disease, (4) severe life-threatening systemic disease, (5) a moribund patient, or (6) a brain-dead organ donor. We also evaluated the degree of independence before the injury, according to the Degree of Independence of Disabled Elderly Persons in Performing Activities of Daily Living criteria proposed by the Ministry of Health and Welfare, Japan [23]. These degrees of independence are classified as Rank 0 = no disability and completely independent in ADL; Rank 1 = some disability, but mostly independent in ADL, and can go outside unassisted; Rank 2 = mostly independent in home-based ADL, but requires assistance to go outside; Rank 3 = requiring considerable assistance in home-based ADL, and uses a wheelchair; Rank 4 = bedridden, requiring assistance for most ADL.

All information on the prescribed oral medications at the time of admission was collected from medical records and patients’ medical notebooks. However, the medicines prescribed for less than 4 weeks, to be taken as needed, and for topical and inhaled use were excluded. 

### 2.4. Outcome Measures

Swallowing abilities were evaluated with the Food Oral Intake Scale (FOIS) [24]; FOIS is scored 1 (poor functioning) to 7 (normal) based on the patients’ ability to intake food and liquids orally. Patients who could intake nothing by mouth were classified into Level 1; those who were tube dependent with minimal attempts of food or liquid were classified into Level 2; those who were tube dependent with consistent oral intake of food or liquid were classified into Level 3; those with a total oral diet of single consistency were classified into Level 4; those with a total oral diet with multiple consistencies, but requiring special preparation or compensations, were classified into Level 5; those with a total oral diet with multiple consistencies without special preparation but with specific food limitations were classified into Level 6; those with a total oral diet with no restrictions were classified into Level 7. The patients with an FOIS level on day 7 and at discharge of ≤5 were diagnosed with dysphagia [1]. 

### 2.5. Statistical Analysis

Continuous and ordinal data are presented as mean ± standard deviations (SD) and the median [25, 75 percentiles], respectively, and the differences were analyzed using the *t*-test and Mann–Whitney *U*-test. Categorical data are expressed as incidents and percentages, with differences analyzed using Fisher’s exact test. Separate analysis for the prevalence of sarcopenia was performed with sarcopenia, low muscle mass (SMI < 5.7 kg/m^2^), low hand-grip strength (<18 kg), and nutritional risk (GRNI ≤ 98). The incidence of dysphagia was also analyzed in different groups classified based on SMI and hand-grip strength. Mean−2SD and mean−4SD values were used as cutoff values for index groups as in earlier studies [25,26]: 4.7 kg/m^2^ and 5.7 kg/m^2^ for skeletal muscle mass [25] and 8 kg and 18 kg for hand-grip strength [26], respectively. The differences were analyzed using Fisher’s exact test. *p*-values < 0.05 were considered statistically significant. The effect size was represented by phi. The statistical tests were performed using SPSS 26.0 software (IBM Japan, Tokyo, Japan).

## 3. Results

**Figure 1:**
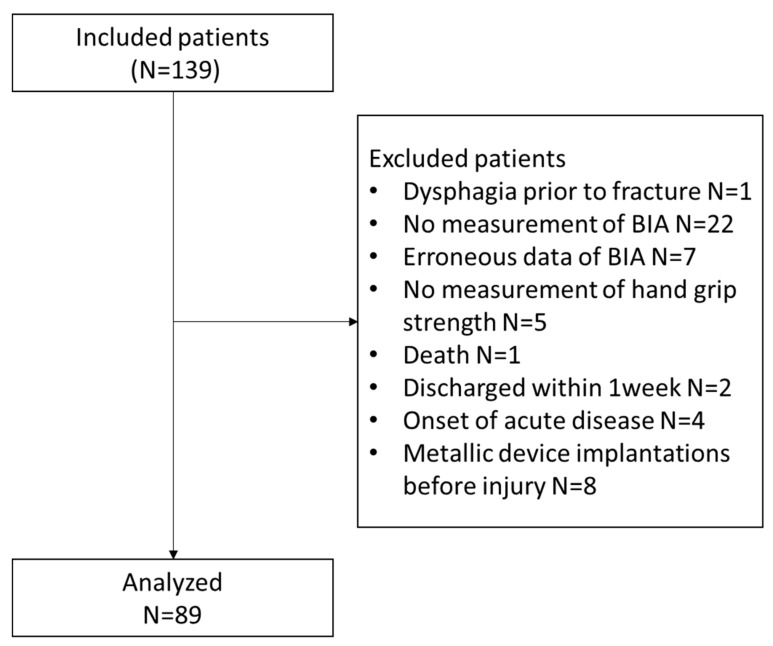
Flow chart for the final study cohort.

In total, 139 patients were admitted to our hospital, of whom 50 were excluded based on the exclusion criteria; finally, 89 patients were analyzed (Figure 1). The mean ages of excluded and included patients were 82.8 ± 10.6 years and 85.9 ± 6.5 years, respectively; the difference in their mean ages was not statistically significant (*p* = 0.089). 

Dysphagia was found in 11 (12.3%) patients on day 7 and 12 (13.5%) patients at discharge. During the study period, no patient developed neurological diseases that could cause dysphagia. The mean length of hospital stay was 20.8 ± 8.1 days. The prevalence of sarcopenia at baseline was 76.4% at baseline. The prevalence of sarcopenia at baseline in patients with dysphagia and without dysphagia on day 7 and at discharge was 100%/73.1% and 100%/72.7%, respectively. The incidence of developing dysphagia among patients with sarcopenia at baseline (N = 68) was 16.2% on day 7 and 17.6% at discharge. GNRI points were significantly low in patients with dysphagia on day 7 and at discharge (*p* = 0.049/0.011). Likewise, both SMI and hand-grip strength were significantly low in patients with dysphagia (*p* = 0.003/0.001 and *p* ≤ 0.001/<0.001), however the prevalence of sarcopenia was not significantly different (*p* = 0.060/0.062). In addition, neither low SMI (*p* = 0.064/0.061) nor low hand-grip strength (*p* = 0.199/ 0.206) was significantly different. Physical dependency and FIM scores were significantly low in dysphagia patients (Table 1). 

The prevalence of dysphagia in different SMI and hand-grip strength groups was analyzed. In the different SMI groups, on day 7, 8 patients (72.7%) and 9 patients (75.0%) at discharge were with low SMI under 4.7 kg/m^2^ at baseline. Patients who developed dysphagia had excessively low SMI at baseline (day 7, *p* = 0.027 and Phi = 0.291; at discharge *p* = 0.012 and Phi = 0.322) (Table 2). In the different hand-grip strength groups: on day 7, 6 patients (40.0%) and 7 patients (75.0%) at discharge had low hand-grip strengths below 8 kg at baseline. Patients who developed dysphagia had excessively low hand-grip strength at baseline (day 7, *p* = 0.003, and Phi = 0.391; at discharge, *p* = 0.001, and Phi = 0.448) (Table 3).

## 4. Discussion

This study was conducted to demonstrate the association between the development of dysphagia after hip fracture surgery in patients with underlying sarcopenia and the prevalence of sarcopenic dysphagia. This present study showed two novel findings. First, all patients who developed dysphagia after surgery had underlying sarcopenia. Second, the prevalence of dysphagia on day 7 and at discharge was 16.4% and 17.4% during the study period, respectively.

Sarcopenia was associated with the occurrence of dysphagia following hip fracture surgery. There was no significant difference in comparison with sarcopenia and low SMI/hand-grip strength defined by AWGS criteria. However, according to subanalysis in the low SMI and hand-grip strength groups, severely low SMI and hand-grip strength were associated with dysphagia development at day 7 and at discharge. No patients had any underlying conditions, such as cardiovascular disease, which could be associated with dysphasia. Therefore, it can be inferred that older people with sarcopenia and severely low SMI and hand-grip strength are at higher risk of developing dysphagia after hip fracture surgery. Previous studies indicated that the risk factors for dysphagia in older patients with hip fracture were low serum albumin level, female gender, mental disorientation following surgery, and reduced physical status (ASA III and IV) [14,15,16,17]. Low serum albumin was associated with sarcopenia [13,27]. 

Some older persons living in care facilities may have lower physical function [28], and deteriorated physical function can be a relevant risk factor for sarcopenia [20]. Furthermore, sarcopenia was associated with the ASA score in patients with hip fractures [13]. Thus, sarcopenia could cause dysphagia in those previous studies, although sarcopenia was not diagnosed in them. Hence, it was ascertained that dysphagia that develops after hip fracture surgery could be sarcopenic dysphagia. Moreover, these patients showed that dysphagia was associated with reduced SMI and hand-grip strength at baseline. This result indicated that worsening sarcopenia was a risk factor for the development of dysphagia following surgical treatment of hip fractures.

In this study, the prevalence of dysphagia after the surgery was 12.3%, which slightly increased to 13.4% at discharge. Previous reports showed the prevalence of dysphagia as 5.3~44% after hip fracture [14,15,16,17]. The difference in the results between those previous studies and ours may be explained by the difference in the diagnosis of dysphagia and the patient characteristics. For instance, Byun et al. assessed the dysphagia using video fluorography and reported its prevalence as 5.3% [17], which may also have been an underestimate because the evaluation was conducted only in patients with possible dysphagia (7.3%). Meals et al. conducted a study on patients undergoing therapies by speech and language pathologists and reported a 42% prevalence of dysphagia [14]; moreover, the survey was conducted in a population suspected of clinical dysphagia, which expectedly reported a higher prevalence of dysphagia. Beric et al. [15] and Love et al. [16] included patients with dysphagia prior to the injury and reported 34% and 44% prevalence of dysphagia, respectively. When Love et al. excluded patients with dysphagia prior to injury in their study [16], the 25.4% patients would have dysphagia after hip fracture, which is close to our result. 

In Japan, the total estimated number of new hip fracture patients in 2012 was about 175,700 in total, about 37,600 for men and about 138,100 for women [29]. Surgical treatments are commonly conducted to treat hip fractures, thus about 17,900 female older patients would possibly develop dysphagia after surgery, assuming the prevalence of developing dysphagia was 13%. In particular, dysphagia is a risk factor for aspiration pneumonia, whose reported prevalence after hip fracture surgery was 4.1~11.1% [30,31,32,33]. Aspiration pneumonia was associated with an increase in mortality [31], readmission rates, and sepsis [32]. In our study, the prevalence of dysphagia after hip fracture surgery was as high as 17.4%. Careful emphasis is required so that clinicians and medical coworkers become aware of the risk of sarcopenic dysphagia after hip fracture surgery and the importance of early detection and preventive intervention for dysphagia. Currently, no established method discusses the prevention or treatment of sarcopenic dysphagia. There are several case reports [9,10,34] and a single interventional study [35] reporting improvement with the treatment of sarcopenic dysphagia. A multimodal approach, including active nutritional therapy and resistance training of the lower extremities, as well as swallowing training and dietary form adjustments, may be effective in the management of sarcopenic dysphagia [34,36]. The rehabilitation nutrition approach [37] is one of the recommended approaches to sarcopenic dysphagia. 

There are several limitations to this study. First, the number of events (the prevalence of dysphagia) was too small to permit a multivariate analysis. Potential confounders such as age and BMI should be adjusted in further studies assessing larger cohorts of patients. Moreover, there are other possible factors associated with sarcopenia development such as Vitamin D and anabolic/catabolic markers, which were not considered in this study [38,39]. Second, the association between sarcopenia and dysphagia and the prevalence of dysphagia in male patients was not clarified since we analyzed only female patients. Third, our study design (single-center study) limits the generalizability of our findings. Further studies on the prevalence of dysphagia after hip fracture surgery and the predictive factors should be conducted on larger cohorts.

## 5. Conclusions

The present study indicates that dysphagia following hip fracture surgery develops in patients with sarcopenia. Progressed sarcopenia was a significant risk for developing dysphagia. Further research studies should determine whether antisarcopenia interventions can prevent the development of sarcopenic dysphagia.

## Figures and Tables

**Table 1:** The baseline characteristics of patients in the study group.

	All N = 89	Day 7			At Discharge		
		Without Dysphagia N = 78	With Dysphagia N = 11	*p*-Value	Without Dysphagia N = 77	With Dysphagia N = 12	*p*-Value
Age, years	85.9 ± 6.5	85.4 ± 6.6	88.9 ± 5.5	0.099	85.5 ± 6.6	88.3 ± 5.6	0.160
Fracture type, n (%)							
Trochanteric	39 (43.8)	36 (46.2)	3 (27.2)	0.335	35 (45.4)	4 (33.3)	0.539
Neck	50 (56.2)	42 (53.8)	8 (72.7)		42 (54.4)	8 (66.7)	
BMI, kg/m^2^	19.6 ± 3.1	19.7 ± 3.2	18.7 ± 2.5	0.334	19.8 ± 3.1	18.0 ± 3.2	0.056
SMI, kg/m^2^	5.0 ± 0.9	5.1 ± 0.9	4.3 ± 0.6	0.003	5.1 ± 0.9	4.3 ± 0.7	0.001
Hand-grip strength, kg	13.2 ± 5.3	14.1 ± 4.9	7.5 ± 4.3	<0.001	14.2 ± 4.8	6.9 ± 3.4	<0.001
Sarcopenia at baseline n (%)	68 (76.4)	57 (73.1)	11 (100.0)	0.060	56 (72.7)	12 (100.0)	0.062
Low SMI at baseline, n (%)	71 (79.8)	60 (76.9)	11 (100.0)	0.064	59 (76.6)	12 (100.0)	0.061
Low hand-grip strength at baseline, n (%)	74 (83.1)	63 (81.0)	11 (100.0)	0.199	62 (80.5)	12 (100.0)	0.206
FOIS at baseline, n (%)							
Level 6	12 (13.5)	6 (7.7)	5 (45.5)	0.004	6 (7.8)	5 (41.7)	0.006
Level 7	77 (86.5)	72 (92.3)	6 (54.5)		71 (92.2)	7 (58.3)	
GNRI, points	91.0 ± 8.9	91.6 ± 8.8	86.0 ± 8.3	0.049	91.8 ± 8.7	84.9 ± 7.7	0.011
Nutritional-related risk							
Low (GNRI: 92 to ≤98), *n* (%)	18 (20.2)	17 (21.8)	1 (9.1)	0.507	18 (23.4)	0 (0.0)	0.069
Moderate (GNRI: 82 to <92), n (%)	36 (40.4)	31 (39.7)	5 (45.4)		30 (39.0)	6 (50.0)	
Major (GNRI: <82), n (%)	13 (14.6)	10 (12.7)	3 (27.3)		9 (11.7)	4 (33.3)	
ASA physical status classification, n (%)							
Class 1	13 (14.6)	12 (15.4)	1 (9.1)	0.550	12 (15.6)	1 (8.3)	0.473
Class 2	12 (15.5)	12 (15.4)	0 (0.0)		12 (15.6)	0 (0.0)	
Class 3	62 (69.7)	52 (66.7)	10 (90.9)		51 (66.2)	11 (91.7)	
Class 4	2 (2.2)	2 (2.6)	0 (0.0)		2 (2.6)	0 (0.0)	
Class 5	0 (0.0)	0 (0.0)	0 (0.0)		0 (0.0)	0 (0.0)	
Physical Dependency Scale before injury, n (%)							
0	30 (33.7)	30 (38.5)	0 (0.0)	0.003	30 (39.0)	0 (0.0)	0.002
1	27 (31.1)	24 (30.8)	3 (27.3)		24 (24.7)	3 (25.0)	
2	22 (24.7)	18 (23.1)	4 (36.4)		17 (22.1)	5 (41.7)	
3	10 (11.1)	6 (7.7)	4 (36.4)		6 (7.8)	4 (33.3)	
4	0 (0.0)	0 (0,0)	0 (0.0)		0 (0.0)	0 (0.0)	
FIM score, points *							
Total FIM	42 (29, 55)	43 (31, 56)	26 (23, 39)	0.007	44 (32, 57)	25 (23, 37)	0.002
Motor FIM	22 (17, 27)	23 (18, 27)	18 (14, 20)	0.019	23 (18, 27)	19 (15, 21)	0.002
Cognitive FIM	19 (11, 31)	20 (12,32)	9 (9,18)	0.016	21 (12, 32)	9 (8, 18)	0.002
The number of medications	5.1 ± 3.5	5.1 ± 3.6	4.8 ± 3.1	0.787	5.2 ± 3.6	4.7 ± 3.0	0.658

Abbreviations: BMI, body mass index; SMI, skeletal muscle mass index; FOIS, Food Oral Intake Scale; GNRI, Geriatric Nutritional Risk Index; ASA, the American Society of Anesthesiologists; FIM, Functional Independence Measure.* 5 data missing.

**Table 2:** Incidence of dysphagia in different groups based on SMI.

	Total N = 89	Dysphagia on Day 7 N = 11	Dysphagia at Discharge N = 12
SMI ≥ 5.7, *n* (%)	18 (20.2) ^a^	0 (0.0) ^b^	0 (0.0) ^c^
4.7 ≤ SMI < 5.7, n (%)	38 (42.7) ^a^	3 (27.3) ^b^	3 (25.0) ^c^
SMI < 4.7, *n* (%)	33 (37.1) ^a^	8 (72.7) ^b^	9 (75.0) ^c^

SMI: skeletal muscle mass index. The number of denominators in the calculation of percentage: a, total; b, dysphagia at day 7; c, dysphagia at discharge.

**Table 3:** Incidence of dysphagia in different groups stratified by hand-grip strengths.

	Total N = 89	Dysphagia at Day 7 N = 11	Dysphagia at Discharge N = 12
Hand-grip strength ≥ 18 kg, n (%)	16 (18.0) ^a^	0 (0.0) ^b^	0 (0.0) ^c^
8 kg ≤ Hand-grip strength < 18 kg, n (%)	58 (65.2) ^a^	5 (9.4) ^b^	5 (9.4) ^c^
Hand-grip strength < 8.0 kg, n (%)	15 (16.8) ^a^	6 (40.0) ^b^	7 (46.6) ^c^

The number of denominators in the calculation of percentage: a, total; b, dysphagia at day 7; c, dysphagia at discharge.
